# Knowledge, Perception, and Management Skills of Mothers with Under-five Children about Diarrhoeal Disease in Indigenous and Resettlement Communities in Assosa District, Western Ethiopia

**Published:** 2015-03

**Authors:** Nigatu Merga, Tadesse Alemayehu

**Affiliations:** ^1^Benishangul Gumuz Regional State Health Bureau, Harar, Ethiopia; ^2^Haramaya University, College of Health and Medical Sciences, Harar, Ethiopia

**Keywords:** Caretaker, Diarrhoea, Knowledge, Management, Mother, Perceptions, Under-five children, Ethiopia

## Abstract

As primary caregiver to under-five children in Ethiopia, mothers’ knowledge, perception, and management skills are important to minimize the effects of morbidity and mortality associated with diarrhoeal diseases. A community-based comparative cross-sectional study was conducted in Abramo and Megele 37 kebeles (the last administration division) in Assosa district of western Ethiopia in July 2010. Quantitative data were obtained by a structured questionnaire from 232 randomly-selected mothers having children aged less than five years regarding their knowledge, perception, and management. Qualitative data were also collected by arranging four focus group discussions involving mothers from the two communities. The prevalence of diarrhoeal diseases among under-five children was 33.2%, and the knowledge of mothers about the causes, transmission, and prevention of diarrhoea in the study area was 37.5%. The prevalence of diarrhoeal disease was higher in the settlement area whereas mothers’ knowledge was better in the indigenous community; 62.9% of mothers were categorized as having good attitude on causes, transmission, and prevention of diarrhoeal disease. Community water source, water storage container, and knowledge of mothers remained a strong predictor of diarrhoeal morbidity after conducting logistic regression analysis (OR=8.4, CI 3.59-31.85; OR=2.2, CI 1.02-4.89; and OR=3.62, CI 1.23-4.71 respectively). Diarrhoeal morbidity was high in the study areas. On the contrary, knowledge and attitude of mothers, recognizing the danger sign of dehydration due to diarrhoea, and the prevention and management of childhood diarrhoeal diseases were not adequate. Information, education and communication strategy may help increase the knowledge and create positive attitude among mothers regarding the cause, prevention, and management of diarrhoea.

## INTRODUCTION

Worldwide, diarrhoea claims the life of 2 million children each year, of which 22% deaths occur in sub-Saharan African countries (1). The burden of diarrhoeal morbidity prevails largely in the developing world where water quality and sanitation and the general living conditions remain poor (2). For example, in Africa, a child below the age of five years experiences five episodes of diarrhoea per year, and 800,000 children die of diarrhoea and dehydration each year (3).

The World Health Organization (WHO) report in 2004 rated Ethiopia as the 4th among the 15 countries with the highest child deaths due to diarrhoea, which was estimated to be 86,000 children (1). The annual report published in 2008 by the Federal Ministry of Health of Ethiopia showed that diarrhoea is the 4th leading cause of morbidity at the national level; in the Benishangul-Gumuz Regional State, diarrhoeal disease was the 2nd leading cause in all health institutions visited (4). As a result, both infant and under-five mortalities are the highest in this region (101 and 169 per 1,000 livebirths respectively) compared to other regions of Ethiopia (5).

In many developing countries, most diarrhoeal episodes are treated at home, and mothers are the key caregivers to under-five children (6). They are the ones who decide about the type of food given to the child and the overall management of the disease. Therefore, their knowledge about this common disease is critically important. Awareness of and perception towards diarrhoea, and individual as well as household actions to prevent and/or manage the disease, have paramount importance to reduce diarrhoea-related morbidities and mortalities (2). On the other hand, mothers’ poor knowledge and attitude about the cause of diarrhoea might limit them from taking appropriate timely actions.

Although the risk factors associated with diarrhoeal disease are theoretically known by programme managers in general terms, empirical evidence is lacking to target the important risk factors specifically that can significantly contribute to its occurrence and distribution in the region in general, and in the current study communities in particular. Factors, such as maternal education, identifying the danger signs, and seriousness of the disease in the indigenous as well as other communities, may also hinder healthy practices in the management of diarrhoeal diseases. This study has assessed the role of knowledge, perception, and practices of mothers about management of diarrhoeal diseases among under-five children in Assosa district of Benishangul-Gumuz Regional State.

## MATERIALS AND METHODS

This comparative cross-sectional study was conducted in Assosa District of Benishangul-Gumuz Regional State in July 2010. Benishangul-Gumuz is one of the nine Regional States in Ethiopia situated in the western part of the country bordering Southern Sudan. This is an area with a year-round evergreen place which attracts many in-country migrants and government-planned settlements. Two rural kebeles (the last administrative division in the country) were purposefully selected from the Assosa district (Figure) to fulfill our purpose of making a comparison. Settlers are living in a few kebeles mostly mixed with the indigenous people. We, however, found two kebeles where the settlers and the indigenous people are living separately (Table 1). The two selected study areas are Abramo (Berta) kebele where indigenous people live and Megele 37 (Amhara) kebele where the settlers are living.

The sampling frame was all households from the two kebeles. We conducted a preliminary listing, and 587 mothers/caregivers with under-five children were identified in the two study kebeles. Sample-size was then calculated using the formula [n=Z^2^ pq/d^2^], assuming the prevalence of diarrhoea in the rural part of the regional state to be 18.5% (5), with 95% confidence interval and 5% margin of error. The calculated sample-size (n=232) was distributed proportionally to the indigenous and settlement communities. Finally, a random-sampling method was applied to select 131 samples from Abramo and 101 from Megele 37.

Quantitative primary data on the basic sociodemographic backgrounds of households and knowledge, perception, and management skills of mothers were gathered using a series of open- and closed-ended structured questionnaire. The questionnaire was administered by face-to-face interview for mothers who had under-five children. To explore the experiences further, their knowledge of and perceptions regarding cause, mode of transmission, prevention, and management of diarrhoeal morbidity, four focus group discussions (FGD) (two from each kebele) were done until saturation level had been reached. The FGD was conducted with mothers who had under-five children and were not taking part in the questionnaire survey. FGD was arranged for women of similar age-groups that could reveal additional interactions and insights, which would be of great interest given the subject under study. Then, the focus group discussants were asked to share their views and personal experiences regarding the causes and prevention of diarrhoeal disease at the household level. We prepared a theme containing five leading questions ahead of the discussion.

**Figure.**
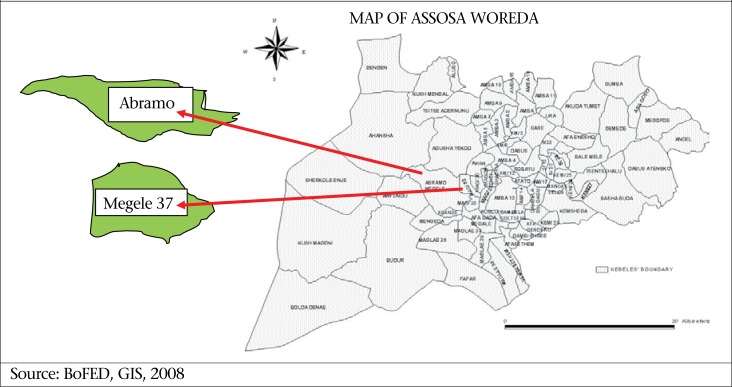
Map of the study area

**Table 1.** Sociodemographic characteristics of mothers with under-five children and community water source in Assosa district, February 2010

Sociodemographic characteristics	Name of kebele				Total	
	Abramo (N=131)		Megele 37 (N=101)			
	No.	Percentage	No.	Percentage	No.	Percentage
Religion of mother						
Muslim	131	100	41	40.6	172	74.14
Orthodox Christian	0	0	60	59.4	60	25.86
Ethnicity						
Berta	131	100	0	0	131	56.47
Amhara	0	0	87	86.14	87	37.5
Oromo	0	0	9	8.9	9	3.88
Tigray	0	0	5	4.95	5	2.16
Family-size						
3-5	66	50.38	54	53.47	120	51.72
6-8	47	35.88	44	43.56	91	39.22
9-12	18	13.74	3	2.97	21	9.05
Educational status of the mother						
Cannot read and write	86	65.65	64	63.37	150	64.66
Can read and write	15	11.45	9	8.91	24	10.34
Elementary school	28	21.37	23	22.77	51	21.98
High school	2	1.53	4	3.96	6	2.59
College and above	0	0	1	0.99	1	0.43
Community water source						
Protected spring	27	10.56	22	16.42	49	16.4
Protected well (hand-pump)	115	70.55	56	41.79	171	57.2
Unprotected spring	1	0.61	9	6.72	10	3.3
Unprotected well	1	0.61	3	2.24	4	1.3
River/Stream	19	10.56	44	32.84	63	21.1

The questionnaire was administered by six 10th grade-completed students who knew the local culture, could speak the two indigenous languages (Berta and Amharic), and had past experience in similar surveys. The data-collectors were trained for two days on how to administer the questionnaire, i.e. one-day in-house training in understanding the questionnaire and one-day field practice in similar communities. Two primary school teachers assisted the principal investigator in conducting FGD. While one of them was interpreting the questions to the participants in the indigenous language, the other was taking detailed notes.

To identify the diarrhoeal cases or the prevalence, diarrhoea was defined as passage of loose stool at least three times a day or more frequent than normal for under-five children in the last 15 days from the time data were collected **(7).** For the purpose of assessing the knowledge of mothers about the cause, transmission, and management of diarrhoeal diseases, five questions were considered for analysis. These questions were: Would you mention some danger signs/symptoms of diarrhoea? Would you mention the cause/mode of transmission of diarrhoea? What did you do when your child got diarrhoea? What are good foods/liquids for a child who has diarrhoea? and What can you do to prevent diarrhoea? As each question contains a multiple correct answers, mothers’ knowledge level for each question was assessed based on how many of them chose correct answers over the total correct choices they should have to answer. After getting the knowledge level for each question, the overall knowledge was calculated from the cumulative percentage of five questions for all respondents.

Attitude of the respondents towards the cause, prevention, and management of diarrhoea was determined using five-point Likert scale. The scale was weighted based on the type of question asked. If the answer was ‘yes’, the responses were coded as 3=strongly agree, 2=agree, 1=neither agree nor disagree, 0=disagree/strongly disagree. If the answer is ‘no’, these codes were reversed. The mean was calculated for each and then for all 12 questions. Finally, those above the mean were categorized as having favourable attitude and below the mean as unfavourable attitude.

The quality of data was assured by pretesting the questionnaire and providing training and strong supervision during data collection. The quantitative data were entered and analyzed using SPSS (version 16.0). Bivariate and multivariable analyses were done to see the association of different factors on the prevalence of diarrhoeal disease. The qualitative data were first transcribed, translated, and then analyzed by identifying themes. The major themes identified during data exploration were causes of diarrhoea, types of diarrhoea, prevention, and common practices (in the community) to treat a child with diarrhoea. Ethical clearance was obtained from the Regional Health Bureau Ethics Committee. Verbal consent was also obtained from each mother/caretaker.

## RESULTS

### Sociodemographic characteristics

All sampled mothers (n=232) participated in the study. The mean age of mothers from both areas was 29.7 years. The mean family-size was 5.7, with interquartile range between 4 and 7. Regarding educational background, 150 (64.7%) could not read and write, and 51 (22.0%) had elementary schooling (Table 1).

### Access to and handling of water for domestic use

People in the study area used water for domestic purpose (drinking, cooking, and personal hygiene) from both protected and unprotected sources. As indicated in Table 1, majority (57.2 %) of the households used water from protected wells whereas only 4 (1.3%) used from unprotected wells (hand-dug well without protective cover made of concrete slab). In general, people in the area depended on multiple sources of water supply because none of the sources was capable to supply their entire water needs throughout the year.

### Knowledge of mothers about diarrhoea

The knowledge of mothers about causes, transmission, and prevention of diarrhoea was found to be 37.5%. The overall knowledge in the indigenous community was 42.67% which was higher than that in the settlement area (32.68%). This result has shown a statistically significant difference (χ^2^=15.915, Df=1, p=0.000) between the two communities. As shown in Table 2, 140 (20.2%) of the respondents reported that diarrhoea can be caused/transmitted by drinking unclean/unsafe water.

### Recognizing the danger signs of diarrhoea

Regarding the danger signs of diarrhoea, 39.5% of the respondents described that passage of three or more loose stools with blood in 24 hours is the immediate danger sign and symptom of diarrhoea. Reported danger sign of diarrhoeal disease was higher in settlement community (51.2%) than the indigenous one (33.8%), although the difference was not statistically significant (χ^2^=0.328, Df=1, p=0.332) (Table 3).

### Recognition and prevention of diarrhoea

Among all respondents, 94.7% mothers from indigenous community and 88.1% of mothers from settlement community reported diarrhoea to be a “serious health problem.” The difference between responses from participants of the two areas was not, however, statistically significant (χ^2^=3.242, Df=1, p=0.06). With multiple responses counted on diarrhoea prevention, 133 (16%) of the responses showed that handwashing after visiting latrine can prevent the occurrence of diarrhoea (Table 4).

**Table 2.** Perceived cause/mode of transmission of diarrhoea reported by mothers with under-five children in Assosa district, February 2010

Cause/mode of transmission of diarrhoea	Abramo (N=493)*		Megele 37 (N=192)*		Total (N=695)*	
	Frequency	%	Frequency	%	Frequency	%
Drinking bad/dirty water	82	16.3	58	30.2	140	20.1
Eating dirty food	79	15.7	39	20.3	118	17
Eating with dirty hands	56	11.1	21	10.9	77	11.1
Eating stale/decayed food	51	10.1	12	6.3	63	9.1
Feeding on dirty utensils	45	8.9	10	5.2	55	7.9
Flies	34	6.8	13	6.85	47	6.8
I do not know	18	3.6	23	11.9	41	5.9
Defaecating in the open field	24	4.8	10	5.2	34	4.9
Eating soil	32	6.3	2	1	34	4.9
Teething	33	6.6	1	0.5	34	4.9
Bottlefeeding	15	2.9	3	1.6	18	2.6
Drinking raw milk	14	2.8	0	0	14	2
Body expelling bad things from stomach	10	1.9	0	0	10	1.4
Drinking raw chicken egg	10	1.9	0	0	10	1.4

*Total number of responses counted (an individual has responded more than once)

**Table 3.** Recognition of danger signs and symptoms of diarrhoea by mothers in Assosa district, February 2010

Variable	Abramo (N=269)		Megele 37 (N=127)		Total (N=390)	
	Frequency	%	Frequency	%	Frequency	%
Passage of >3 loose stools with blood in 24 hours	89	33.8	65	51.2	154	39.5
Thirst and dry mouth	50	19	7	5.5	57	14.6
Sunken eyeballs	46	17.5	8	6.3	54	13.8
Tearless eyes	34	12.9	1	0.8	35	9
Loss of stretchiness of the skin	29	11	26	20.5	55	14.1
I do not know	15	5.7	20	15.7	35	9

### Management of diarrhoeal disease

Majority (62.4%) of the mothers reported that they sought treatment from licensed medical practitioners for their children while s/he had diarrhoea. Only 21 (9%) of them reported that they took to the traditional healers. Giving oral rehydration salts solution (ORS) was reported by 48 (13.7%) mothers during such condition. A statistically significant difference (χ^2^=8.45, Df=1, p=0.003) was seen as 10.2% of mothers from the settlement area and 15.5% from the indigenous community reported giving ORS. As home-remedy, 30 (8.5%) of mothers said that they gave homemade fluids, and 18 (5.1%) of mothers fed breastmilk during diarrhoeal episode. To the contrary, 9 (2.6%) mothers reported that they stopped feeding liquid-containing food items (including milk and homemade fluids) when their child had diarrhoea (Table 4).

### Attitudes of the mothers towards diarrhoea

Based on the analysis of attitude-related questions, 146 (62.9%) of mothers were categorized as having favourable attitude (Table 5). This study found that mothers had different attitudes/beliefs about transmission of diarrhoeal disease, prevention and control methods. For instance, 70.7**%** of the respondents identified ‘teething’ as one of the major causes of diarrhoea.

In response to the statement “diarrhoea attacks mostly bottlefed children”, 54.7% of mothers disagreed. Among 38.8% of the mothers who agreed to the statement “liquid food aggravates diarrhoea”; considerable numbers of mothers fed their child dry food items: 12.2% fed locally-prepared bread (*kita*), coffee powder (9.8%), and roasted corn (5.9%) while s/he had diarrhoea.

**Table 4.** Reported methods of diarrhoea prevention and management by mothers with under-five children in Assosa district, February 2010

Variable	Abramo (N=587)*		Megele 37 (N=243)*		Total (N=830)*		Chi-square statistics (χ^2^, p value)
	Frequency	%	Frequency	%	Frequency	%	
Method for prevention of diarrhoea							
Washing hands after visiting latrine	90	15.3	43	17.7	133	16	χ^2^=15.92, p=0.000
Washing hands before food preparation	68	11.6	29	11.9	97	11.7	χ ^2^=12.6, p=0.000
Washing hands after handling child's faeces	67	11.4	27	11.1	94	11.3	χ^2^=14.1, p=0.000
Drinking clean water	55	9.4	31	12.8	86	10.4	χ^2^=3.12, p=0.051
Wash hands before feeding a child	62	10.6	13	5.3	75	9	χ^2^=30.95, p=0.00
Wash hands before eating	50	8.5	19	7.8	69	8.3	χ^2^=10.2, p=0.001
Handle food hygienically	33	5.6	23	9.5	56	6.7	χ^2^=0.18, p=0.39
Using latrine for defaecation	36	6.1	17	7	53	6.4	χ^2^=3.67, p=0.038
Store water in clean container	38	6.5	11	4.5	49	5.9	χ^2^=11.24, P=0.001
Disposing children's faeces into toilet	38	6.5	10	4.1	48	5.8	χ^2^=12.69, P=0.00
Treating water with chemicals/chlorine	38	6.5	9	3.7	47	5.7	χ^2^=14.26, p=0.000
I do not know	12	2	11	4.5	23	2.8	χ^2^=0.19, p=0.41
Management of diarrhoea							
Seek modern treatment	123	52.8	96	81.4	219	62.4	χ ^2^=0.14, p=0.67
Giving ORS	36	15.5	12	10.2	48	13.7	χ ^2^=8.45, p=0.003
Feeding breastmilk	16	6.9	2	1.7	18	5.1	χ ^2^=8.35, p=0.003
Giving homemade fluids	24	10.3	6	5.1	30	8.5	χ ^2^=7.76, p=0.003
Stop feeding	8	3.4	1	0.8	9	2.6	χ ^2^=4.0, p=0.043
Take to traditional healers	21	9	0	0	21	6	χ ^2^=17.8, p=0.000
Do not know	5	2.1	1	0.8	6	1.7	χ ^2^=1.81, p= 0.18

*Total number of responses counted (an individual has responded more than once)

**Table 5.** Mothers’/caretakers’ attitude towards diarrhoea in Assosa district, February 2010 (N=232)

Attitude item	Statistic		
	Mean	>Mean (%)	<Mean (%)
Diarrhoea attacks mostly bottlefed children	2.86	96 (41.4)	136 (58.6)
Diarrhoea is a disease of the poor	2.41	55 (23.3)	177 (76.3)
Diarrhoea is a killer disease	3.88	192 (82.8)	40 (17.2)
Diarrhoea is a problem in the community	3.66	184 (79.3)	48 (20.7)
Teething causes diarrhoea	3.52	164 (70.7)	68 (29.3)
Diarrhoea is a curable disease	3.72	185 (79.7)	47 (20.3)
Child's/infant's faeces are not hazardous to health	2.43	56 (24.1)	176 (75.9)
Liquid food aggravates diarrhoea	2.75	90 (38.8)	142 (61.2)
It is important to continue feeding breastmilk when a child has diarrhoea	3.68	177 (76.3)	55 (23.7)
Oral rehydration salts solution cures diarrhoea	3.59	169 (72.8)	63 (27.2)
Human faeces are a source of diarrhoea	3.84	189 (81.5)	43 (18.5)
Handwashing prevents diarrhoea	3.77	190 (81.9)	42 (18.1)
Total score	2.63	146 (62.9)	86 (37.1)

### Prevalence of diarrhoea and its risk factors

Among the participant mothers, one-third (33.2%) reported that their child had diarrhoea during the past two weeks. The prevalence was higher in the settlement area (71.9%) than in the indigenous community (29%). When we examined the relationship between different factors with prevalence of diarrhoea, recognizing diarrhoea as a serious health problem, community water source, placement of water-storage container, availability of latrine, and knowledge and attitude of mothers were found to be statistically associated (Table 6).

In multiple logistic regression analysis, only water source for the communities, placement of water-storage container, and knowledge of mothers showed a strong statistically significant association with prevalence of diarrhoea in the last two weeks. Under-five children using water from unprotected sources had more than 8 times higher odds of having diarrhoea than those from protected water sources (OR=8.4, CI 3.59-31.85, p=0.000). Children from mothers who had low knowledge about diarrhoeal disease had 3.6 times higher odds of getting diarrhoea than their counterparts (OR=3.62, CI 1.23-4.71, p=0.001). At the same time, those who did not place water containers raised from the floor had 2.2 times higher odds of getting diarrhoea in under-five children than those who raised the water container from the floor (OR=2.2, CI 1.02-4.89, p=0.044).

### Focus group discussion

As causes of diarrhoea, all the participants in focus group discussion (FGD) mentioned different factors but no one mentioned more than two factors. During the discussion, participants reflected similar view about the cause and transmission of diarrhoea, which was consistent with the quantitative data. For example, more than half of the FGD participants mentioned teething as the main cause of diarrhoea. At the same time, many of them listed contaminated water and food, flies, and faecal materials as the ways of transmission of diarrhoeal diseases.

Apart from those who reported that they took their child to health facility, traditional healer, gave ORS, many of the FGD participants mentioned some other alternative treatments. A 36-year old mother from settlement community said, “I gave dry food, like bread, to stop diarrhoea.” Another 24-year old woman from the same kebele reported, “Coffee powder mixed with honey is good for diarrhoea patient because it stops diarrhoea immediately.” Two mothers from Berta/indigenous community said, ‘We gave homemade fluids, like porridge, roasted corns, lemon juice, garlic, water, and tea, to the patient to replace the fluid taken out from the body.”

A 28-year old mother from Megele 37 kebele said, “I gave my child *Arakie* (local alcohol drink) while having diarrhoea in order to kill the germ/bacteria that causes diarrhoea in the stomach. This is what I have got through my life experience. This practice is exercised by many mothers in my locality. When a patient drinks the alcohol, his health improves.”

## DISCUSSION

The findings of this study showed that the prevalence of diarrhoea for a two-week period among under-five children was 33.2%. Children from the settlement kebele were more affected than from the indigenous (Berta) community (χ^2^=2.322, Df=1, p=0.012). This figure is similar to the result of study done in Nekemte town of Ethiopia (28.9%) (8). However, it is quite higher compared to the prevalence of diarrhoea in western Ethiopia (18%), in Keffa-Sheka Zone of Southwest Ethiopia (15%), and northern part of Ethiopia (17.9%) (9–11). The differences in prevalence might be attributed to the representativeness of the sample and the differences in the study set-up. The low prevalence of diarrhoea in the indigenous community could be because of the exhibited better knowledge of mothers in this community.

**Table 6.** Factors relating to diarrhoeal morbidity in the two study areas of Assosa district, February 2010

Variable	Had the child have diarrhoea in the past 15 days?		AOR	95% CI	p value
	Yes	No			
Do you recognize that diarrhoea is a serious health problem?					
Yes	74	139	0.32	0.08-9.94	0.04
No	3	16	1.0		
Community					
Abramo (Berta)	38	37	0.48	0.15-0.95	0.011
Megele 37 (Amara)	93	62	1.0		
Water sources for community					
Protected source	26	138	1.0		
Unprotected source	39	9	8.4	3.59-31.85	0.000
Water-storage container placed/raised from the floor					
Yes	35	98	1.0		
No	42	57	2.2	1.02-4.89	0.008
Items present for handwashing					
Yes	20	56	1.0		
No	48	91	1.1	0.43-2.77	0.94
Availability of latrine					
Yes	9	8	1.0		
No	68	147	1.3	1.16-11.18	0.026
Educational level of mothers					
Non-literate	52	98	3.1	0.61-5.8	0.31
Literate	25	57	1.0		
Knowledge level of mothers					
Knowledgeable	91	55	1.0		
Not knowledgeable	53	33	3.62	1.23-4.71	0.001
Attitude of mothers					
Good attitude	92	54	1.0		
Poor attitude	54	32	1.25	0.45-1.99	0.065

AOR=Adjusted odds ratio

Mothers’ knowledge level regarding the cause/transmission of diarrhoea by drinking unclean/unsafe water is found to be low (20.15%) compared to other studies (2,12). For example, a study conducted in Malawi (12) showed that 55% informants and, in another study in rural community of Kenya **(2),** 58.2% reported contaminated water as principal cause of diarrhoea. Such disparity might be due to difference in educational background between the women in Malawian study and women in our study. The Malawian study (12) showed that 79% of women attended elementary school and above whereas only 25% of mothers in our study attended schooling at the same level.

Above one-third of the respondents (39.5%) mentioned that passage of three or more loose stools with blood in 24 hours is the immediate danger sign of diarrhoea. This result is contrary to the findings of a study done in rural community of Kenya, which reported 76.4% of mothers/caretakers were not able to mention any of the danger signs (2). Having a good knowledge in one of the pertinent danger signs of diarrhoeal diseases by caregivers is important as Othero and colleagues in rural community of Kenya (2) had identified that knowledge of danger signs is important as early referral of very sick children is necessary for appropriate treatment. However, failing to identify key danger signs and taking prompt actions may result in major complications or death. On the other hand, the finding of our study suggested that there is association between knowledge of mothers about the cause, transmission, and prevention of diarrhoeal disease and prevalence of diarrhoea (OR=3.62, CI 1.23-4.71, p<0.001). Other studies (13,14) also showed that mothers with low knowledge had association with incidence of children's diarrhoea.

More mothers from the indigenous community agreed with the statement “teething causes diarrhoea” than their counterparts in the settlement community. The relation between teething and diarrhoea is significantly different between the two communities (χ^2^=13.005, Df=1, p=0.001). This shows that mothers in the indigenous community had more misconception about the relation between teething and diarrhoea. The relationship between teething and diarrhoea is also a common misconception in other parts of the world. In Enugu state of Nigeria, about 69.8% to 71.9% of mothers perceived teething as a major cause of diarrhoea (15). Another study conducted in Kingston, Jamaica, showed that 82% of the Jamaican caregivers thought teething water could cause diarrhoea (14).

According to the community integrated management of childhood illness strategy (16), mothers/caregivers at home should have adequate knowledge on the causes and prevention as well as treatment of diarrhoea, using appropriate remedies, including homemade fluids, such as fresh fruit juices, milk, salt/water solution, and breastmilk. In addition, mothers/caretakers should seek treatment for their child from health facilities to limit any damage on the child's health that may have been caused by diarrhoeal disease (16). From this point of view, our study revealed that 62.4% of mothers/caretakers seek treatment from licensed medical practitioners for their child who gets diarrhoea, and this practice is similar to those observed in the study done in rural community of Kenya (2). As the result of this study revealed, there are still considerable number of mothers who prefer other options to taking the sick child to health institution, which, to some extent, may predispose the health of the child into great danger. Identifying such malpractices and working towards tackling their impact will help the Government to reduce child mortality, one of the key health problems in the country. The county is showing relentless efforts to reduce child mortality and morbidity where managing diarrhoeal disease plays a big role.

Education of mothers/caretakers plays an important role in the prevention of diarrhoeal diseases and for caring a sick child (9,13). Mothers with higher education are thought to have better opportunity for information about childcare than mothers/caretakers with lower educational level (17). Accordingly, our study revealed that there was higher prevalence of diarrhoea (67.5%) in the households whose mothers cannot read and write, although it was not statistically significant. Mothers with less educational status may not have basic knowledge on the impacts of potential risk factors, such as water supply, latrine utilization, hygiene, and sanitation, on the occurrence of diarrhoea (12). This finding is consistent with a finding in Zimbabwe (18).

Age of the child was another factor associated with prevalence of diarrhoea among under-five children in our study. Children in our study with age of less than two years were more affected than others (χ^2^=10.124, Df=3, p<0.01), which are consistent with other studies (13,18). The possible reason for this high prevalence is that children aged less than two years have less immunity against any disease and could be easily susceptible to infection than older ones (19). Children are also at greater risk than adults of life-threatening dehydration since water constitutes the largest proportion of children's body-weight (6).

Our study showed a significant association between unprotected source of drinking-water and diarrhoeal morbidity among under-five children (CI 3.59-31.85, p<0.000). This finding was consistent with the study conducted in Uganda, which shows that there was strong association between source of drinking-water obtained from rivers/streams and diarrhoeal morbidity among under-five children (p<0.001) (13).

### Limitations

Some of the risk factors of the prevalence of diarrhoea found in our study were not significantly associated with childhood diarrhoea. This might be due to the small sample-size in this study, which underestimates the effect of those risk factors on childhood diarrhoeal morbidity (18).

### Conclusions

The findings of this study showed that the prevalence of diarrhoea for a two-week period preceding the study in under-five children is found to be very high. On the other hand, the knowledge of mothers on perceived cause, recognition of danger signs, prevention, and management of diarrhoea is inadequate.

The knowledge of mothers was found to be essential for reducing occurrence of diarrhoea for under-five children. On the other hand, considerable number of mothers from both indigenous and settlement communities had negative attitudes in some aspects of the cause, transmission, and management of diarrhoeal disease. The gaps existing among mothers in understanding management of diarrhoea needs to be addressed by proper information, education and communication activities.

### Recommendations

Early identification of danger signs and treatment and management of diarrhoea at home is essential to prevent deaths due to dehydration. Preventive efforts to control diarrhoeal diseases should focus on increasing the level of knowledge and attitude of mothers/caregivers and on addressing the behavioural factors in relation to management of diarrhoea. As the prevalence of diarrhoea was found to be high, health professionals, together with community health workers, should perform surveillance activities for early detection of the problem and addressing the risk factors associated with the disease. Health education, community conversation, dissemination of information, and communication skills should be planned and implemented to increase the knowledge and to minimize the misconceptions of the mothers/caretakers as well as the community as a whole.

## ACKNOWLEDGEMENTS

Authors of this paper would like to thank the respondents for their willingness to participate in the study and provide valuable information. We also extend our gratitude to our data-collectors for their willingness to participate, commitment and enduring hardships, and challenges during the whole period of data collection. Our acknowledgement extends to the Health Bureau of Benishangul-Gumuz Regional State for funding this study.

**Conflict of interest:** Authors declare no competing interests.

## References

[1] Boschi-Pinto C, Velebit L, Shibuya K (2008). Estimating child mortality due to diarrhoea in developing countries. Bull World Health Organ.

[2] Othero DM, Orago AS, Groenewegen T, Kaseje DO, Otengah PA (2008). Home management of diarrhea among underfives in a rural community in Kenya: household perceptions and practices. East Afr J Public Health.

[3] Woldemicael G (2001). Diarrhoeal morbidity among young children in Eritrea: environmental and socioeconomic determinants. J Health Popul Nutr.

[4] Ethiopia. Ministry of Health (2007). Health and health related indicator.

[5] Ethiopia. Central Statistical Agency (2012). Ethiopia demographic and health survey 2011.

[6] Ghasemi AA, Talebian A, Masoudi Alavi N, Mousavi GA (2013). Knowledge of mothers in management of diarrhea in underfive children, in Kashan, Iran. Nurs Midwifery Stud.

[7] The United Nations Children's Fund (2009). Diarrhoea: why children are still dying and what can be done.

[8] Eshete WB (2008). A stepwise regression analysis on under-five diarrhoael morbidity prevalence in Nekemte town, western Ethiopia: maternal care giving and hygiene behavioral determinants. East Afr J Public Health.

[9] Dessalegn M, Kumie A, Tefera W (2011). Predictors of under-five childhood diarrhea: Mecha District, West Gojam, Ethiopia. Ethiop J Health Dev.

[10] Teklemariam S, Getaneh T, Bekele F (2000). Environmental determinants of diarrheal morbidity in under-five children, Keffa-Sheka zone, south west Ethiopia. Ethiop Med J.

[11] Mitike G (2001). Prevalence of acute and persistent diarrhoea in north Gondar zone, Ethiopia. East Afr Med J.

[12] Masangwi SJ, Grimason AM, Morse TD, Kazembe L, Ferguson N, Jabu GC (2012). Pattern of maternal knowledge and its implications for diarrhoea control in Southern Malawi: multilevel thresholds of change analysis. Int J Environ Res Public Health.

[13] Mbonye AK (2004). Risk factors for diarrhoea and upper respiratory tract infections among children in a rural area of Uganda. J Health Popul Nutr.

[14] Bachrach LR, Gardner JM (2002). Caregiver knowledge, attitudes, and practices regarding childhood diarrhea and dehydration in Kingston, Jamaica. Publica.

[15] Ene-Obong HN, Iroegbu CU, Uwaegbute AC (2000). Perceived causes and management of diarrhoea in young children by market women in Enugu State, Nigeria. J Health Popul Nutr.

[16] WHO/UNICEF (2002). Integrated management of childhood illness information.

[17] Yilgwan CS, Okolo SN (2012). Prevalence of diarrhea disease and risk factors in Jos University Teaching Hospital, Nigeria. Ann Afr Med.

[18] Root GP (2001). Sanitation, community environments, and childhood diarrhoea in rural Zimbabwe. J Health Popul Nutr.

[19] Maneekarn N, Ushijima H (2000). Epidemiology of rotavirus infection in Thailand. Pediatr Int.

